# Does gaze direction influence cycling effort?

**DOI:** 10.1371/journal.pone.0327377

**Published:** 2025-07-02

**Authors:** Sem Otten, Ruud J. R. Den Hartigh, Frank T. J. M. Zaal, Benoît G. Bardy, Christophe Gernigon

**Affiliations:** 1 EuroMov Digital Health in Motion, University of Montpellier, IMT Mines Alès, Montpellier, France; 2 Department of Psychology, University of Groningen, Groningen, The Netherlands; 3 Center for Human Movement Sciences, University of Groningen, University Medical Center Groningen, Groningen, The Netherlands; University of Giessen: Justus-Liebig-Universitat Giessen, GERMANY

## Abstract

Cycling effort can be influenced by the speed of the optic flow to which individuals are exposed. The present study tested whether gazing toward proximal (e.g., the road in front) versus distal areas (e.g., the horizon ahead) would influence cycling effort. We expected that gazing toward proximal areas would generate a feeling of “momentum” and thereby increase efforts. Twenty-eight cyclists completed two 20-minute trials on their bicycle in a VR environment, aiming to outperform the power output they exerted during a baseline trial. Their gaze direction was guided through a virtual frame, either aimed at the road immediately in front of the cyclist (proximal) or at the horizon (distal), in counterbalanced order. A repeated measures ANCOVA, with baseline power as a covariate, showed no significant difference in exerted effort between the proximal and distal conditions, and no significant interaction effect between condition and baseline power. This finding is not in accordance with previous research, in which occlusions of proximal and distal areas of the visual field did influence cycling efforts. Taken together, the results suggest the importance of peripheral vision in speed perception, which may influence cycling effort.

## Does gaze direction influence cycling effort?

Effort regulation during exercise emerges from a complex interaction between physiological, psychological, and environmental factors [1]. This regulation involves both feedforward signals from higher brain centers, as well as bottom-up sensory feedback from cardiovascular, respiratory, and musculoskeletal systems [2,3]. Among these various influences, vision has also been shown to be part of effort regulation. That is, optic flow – the structural visual motion generated by an individual’s movement through the environment – has been shown to influence how cyclists perceive and regulate their effort [4]. Interestingly, the velocity of elements in the optic flow of a moving observer is not uniform across the optic flow field. Optic flow related to proximal areas in the environment, such as the road directly in front of a cyclist, contains faster-flowing elements, while distal areas, such as those near the horizon, contain slower-flowing elements [5]. As a result, exposing cyclists to proximal areas of the environment exposes them to faster flowing elements than exposing them to distal areas, thereby possibly increasing speed perception [6] and exerted effort [7].

Recently, a first experimental study was conducted on the effect of occluding proximal and distal areas on exerted effort during virtual reality (VR) cycling trials [7]. The study found that exerted cycling effort was influenced by proximal and distal exposure, with the relationship depending on cyclists’ baseline power. Specifically, cyclists with lower baseline power exerted significantly more effort when only proximal areas were shown compared to when only distal areas were shown. However, this effect diminished as baseline power increased, and for cyclists with the highest baseline power, the pattern reversed. These cyclists exerted significantly more effort under the distal condition.

This finding may have practical implications for cyclists, as it suggests that directing their gaze toward proximal or distal areas could influence their exerted effort. For example, lower-level cyclists could potentially enhance their performance by gazing on the road directly in front of them during time trials. However, given that cyclists cannot ride with parts of their visual field occluded, it remains to be tested whether simply guiding their gazing without any occlusion would yield similar effects. The current study aims to investigate the effects of such gaze strategies on cycling effort without using occlusion. In the following sections, we will first explore how the optic flow related to proximal and distal areas of the environment can influence exerted effort. We will then discuss the potential effects of guided proximal and distal gazing on cycling effort, which we will test empirically.

### The role of optic flow in cycling effort

Parry et al. [4] were the first to demonstrate the relationship between optic flow velocity and effort in cycling trials. In their study, cyclists performed virtual trials in which optic flow was manipulated to unfold either faster, slower, or at the same speed as when riding at their actual cycling speed, while they were tasked with matching their power output and cadence to a baseline trial that they had performed earlier. When optic flow was slowed down, cyclists reported lower ratings of perceived exertion (RPE) [8] but exerted significantly more effort compared to a situation in which optic flow matched their actual speed or was sped up. These findings suggest that slower optic flow elicits a response in which cyclists increase their effort as a direct adjustment to the decreased optic flow velocity [4]. This aligns with a broader body of research, which shows a general negative association between experimental increases or decreases in optic flow velocity, and energy expenditure or walking speed [9–17].

In the above-mentioned studies, optic flow is often manipulated by slowing down or speeding up the entire optic flow field. However, flow velocities vary across different areas of the visual field [5]. For instance, while moving, an observer’s optic flow is characterized by faster velocities related to proximal areas and slower velocities related to distal areas of the environment. This means that during cycling, gazing at the road nearby exposes cyclists to higher flow velocities than gazing farther ahead. Given this, Otten et al. [7] explored whether exposure to proximal or distal areas, through occlusion of the other areas, would also lead to differences in cycling effort. They designed an experiment in which cyclists first completed a baseline trial, during which they were instructed to produce maximum power while viewing a complete virtual environment. The average power output from this trial was recorded, and in two subsequent experimental sessions, the cyclists were instructed to outperform their baseline power output with parts of their visual field occluded: One session with only the proximal area exposed and the other with only the distal area exposed.

The authors hypothesized that exposure to a proximal area, with its faster-flowing elements, would enhance cyclists’ sense of goal progress (i.e., beating one’s own baseline power) [7]. This sense of progress is a characteristic of psychological momentum (PM) and has been linked to increased effort during goal pursuit [18–20]. Based on this, they predicted that cyclists would exert more effort during proximal exposure. Partly consistent with this hypothesis, their findings showed that cyclists exerted more effort during exposure to the proximal area. However, this effect was dependent on baseline power levels. Cyclists with relatively low baseline power exerted more effort during proximal exposure, but this effect disappeared as baseline power increased and even reversed for cyclists with the highest baseline power, who exerted more effort under distal exposure [7].

Despite the increased effort observed during proximal exposure, Otten et al. found no evidence that this effect was associated with heightened PM experiences. Throughout the trial, responses to the statement “I feel like I’m progressing towards my goal”, which was used to measure PM, did not significantly differ between the proximal and distal conditions, nor did these responses correlate with exerted effort. This discrepancy may be explained by the complex nature of PM, which involves cognitive, affective, motivational, physiological, and behavioral dimensions, making it difficult to capture with a single question [19–22]. Additionally, as the results were in the expected direction, with higher PM experience reported in the proximal condition, the authors suggested that momentum-like responses may still have been present, even if the cyclists were unable to fully express them through the PM item they responded to.

### The role of peripheral vision in speed perception and cycling effort

By occluding proximal and distal areas, Otten et al. [7] effectively reduced participants’ field of view (FoV). Research has consistently shown that reducing FoV size can lead to underestimations of speed [23–27]. For example, in cycling simulations, speed was underestimated with a narrower FoV and overestimated when the FoV was expanded [27]. Similarly, Lidestam et al. [23] found that expanding the FoV both horizontally and vertically into the peripheral areas in driving simulations resulted in faster perceived speed, as reflected in lower driving speeds.

Speed perception also depends on which area of the FoV is reduced. Pretto et al. [25] showed that when central areas were occluded (i.e., areas near the focus of expansion, where all motion vectors expand and towards which the observer is moving), perceived speed increased. Conversely, occluding peripheral areas (i.e., areas far from the focus of expansion) led to decreased perceived speed. This may be explained by the fact that peripheral areas, farther away from the focus of expansion, contain faster flowing elements than central areas near the focus of expansion [5].

While these studies show that occluding areas of the visual field has clear effects on speed perception, it is less well understood how gaze direction might influence similar processes when the entire optic flow field remains visible. This question is particularly relevant given the peripheral retina’s ability to process motion effectively. Unlike spatial resolution and color sensitivity, which decline significantly farther away from the fovea, motion sensitivity remains relatively robust in the peripheral retina [28,29]. That is, when gazing at central areas, a moving observer can still process motion in peripheral areas, which may influence perceived speed and effort, and thereby exerted effort.

This begs the question whether the differences in exerted cycling effort observed by Otten et al. [7], elicited through proximal or distal exposure, could be replicated if occlusion is replaced with directed gazing alone. One study suggests that gaze direction itself can, indeed, affect speed perception independently of FoV size. In this study, Banton et al. [6] showed that walkers perceived a 4.8 km/h walking speed to be about 50% slower when gazing straight ahead compared to when gazing downward. This suggests that the influence of optic flow exposure on speed perception may not be solely due to reducing the FoV but could also occur when gaze direction is altered.

### The present study

With the present study we aimed to test whether guiding cyclists’ gaze toward proximal or distal areas affects effort during cycling trials. Building on previous findings that cycling effort was influenced by occluding proximal and distal areas, we investigated the effects of gazing at proximal or distal areas in a VR environment without occluding other areas. By allowing both proximal and distal areas to remain in peripheral vision, this study thereby aimed to replicate and extend previous findings in a more naturalistic setup. Overall, we hypothesized that gazing at proximal areas would trigger more positive PM, thereby leading to greater effort compared to gazing at distal areas. Given that previous research found that this effect was qualified by baseline power, we explored whether baseline power would play a moderating role in the current study as well.

## Materials and methods

### Participants

We performed a sample size calculation using G*Power (3.1.9.7) based on a repeated measures ANOVA with two conditions (proximal vs. distal). The calculation indicated that a minimum of 34 participants would be required to achieve a statistical power of 0.8, assuming a medium effect size (*f* = 0.25) and an alpha level of.05. Although Otten et al. [7] reported a large effect of the proximal and distal exposure conditions on exerted effort (*f* = 0.56), the current study allowed full peripheral vision and was expected to produce a smaller effect. We therefore used a more conservative, medium effect size for the power analysis. We recruited 34 cyclists, aged 18–50, who were accustomed to performing cycling trials and had normal or corrected-to-normal vision. Recruitment was conducted through online platforms, university faculties, and cycling clubs between 10/01/2024 and 10/06/2024. Five participants did not complete all trials and were excluded, while one participant revealed after the final trial that he had held back during the proximal trial due to an important match the following weekend, leading to his exclusion as well. As a result, the final sample consisted of 28 participants (18 male, 10 female; mean age = 26.5 years, *SD* = 6.3; mean height = 1.81 m, *SD* = 0.08; mean weight = 73.5 kg, *SD* = 10.1). On average, participants had 6.95 years of road cycling experience (*SD* = 6.23), cycled a minimum of once per week, with a median of 2 sessions per week (IQR 1.00–3.00), and trained a median of 4.50 hours weekly (IQR 2.13–7.75). Participants provided written informed consent before participating and received a financial reward. The study was approved by the Ethics Committee of the Faculty of Behavioural and Social Sciences (EC-BSS: PSY-2324-S-0084) at the University of Groningen.

### Materials

Participants used their own bicycle, mounted on a Tacx NEO T2 Smart trainer (Wassenaar, The Netherlands), to perform a baseline trial and two “gazing” trials (proximal and distal). The device recorded instantaneous power output in Watts with a deviation of less than 1% and simulated a horizontal ride with a consistent 0% slope throughout the trials. Manual gear adjustments were allowed during the trials.

The virtual environment was displayed using an HTC Vive Pro Eye head-mounted display (HMD) which has a 110° diagonal FoV and a 1440x1600 per-eye resolution. The virtual environment ran on a Windows 10 computer with a 64-bit operating system, equipped with an Intel Core i7-4770 processor (3.4 GHz), 32 GB of RAM, and an NVIDIA GeForce GTX 1650 graphics card. The virtual environment was created using Unity 2019.4.36f and depicted a straight and flat road running through a mountainous landscape with occasional roadside elements such as trees and rocks. A timer above the road indicated the remaining trial time. Power output from the Tacx was coupled via Bluetooth to the virtual landscape’s velocity, creating a sense of self-motion consistent with real-world cycling dynamics. The Tacx sent power output data at approximately 2.4 Hz to a local Python script, which was continuously read by Unity to update the bicycle’s movement. This pipeline introduced an estimated delay of 450–500 ms between cycling effort and movement through the virtual scene. Pilot testing indicated that the delay was mainly noticeable at the onset of pedaling, before the trial started, and did not affect the natural feel of movement once the trial was underway.

**Fig 1:**
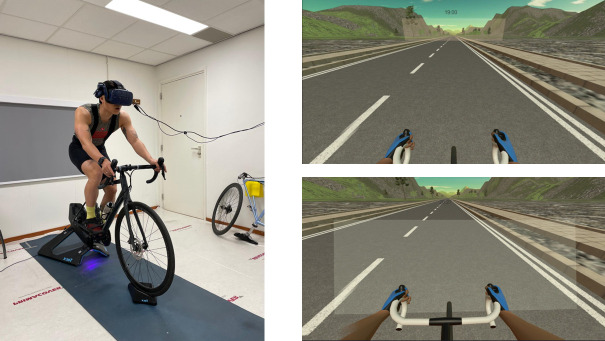
Research setup and view of the gazing frames in the distal condition (top right) and proximal condition (bottom right).

During the gazing trials, a virtual frame was placed in either the proximal or distal area of the cyclists’ view. The distal frame focused on the horizon, while the proximal frame focused on the road immediately in front of the cyclist (see Fig 1). When the proximal frame was displayed, the timer was placed more downwards to be in the cyclist’s view. Every 2 minutes, an RPE (Borg CR10) [30,31] and a PM item appeared in the cyclist’s view. The PM item was a direct measure of psychological momentum, displaying the statement “*At this moment, I have the momentum*” rated on a scale from 0 (*not at all*) to 10 (*very strongly*). We adapted the PM item from Vallerand et al. [32], changing the original wording from “Who has the momentum?” to “At this moment, I have the momentum” to better suit our individual cycling task. We also modified the original 1-to-7 scale to a 0-to-10 scale to align with the Borg CR10. After responding, the items disappeared from view until the next assessment. These measures were taken to explore any differences in perceived effort and PM between the two conditions, and whether PM related to exerted effort

We recorded participants’ gaze direction throughout the trials using a Tobii XR eye-tracker (Core SW 2.16.4.67) embedded in the HMD, with a sampling frequency of 120 Hz (HTC Corporation, Taoyuan, Taiwan). We accessed the eye-tracking data within the Unity project using Vive SRanipal SDK v1.1.0.1 (HTC Corporation, Taoyuan, Taiwan) and Unity’s raycasting method [33]. This method involves projecting an invisible ray from the gaze origin (i.e., the 3D position representing the participant’s eyes) in the direction of the participant’s gaze. When the ray intersects with a virtual object, the system registers the three-dimensional coordinates of the intersection point (i.e., the hitpoint) within the virtual environment. These hitpoint coordinates were used to calculate the average vertical and longitudinal gaze positions, which served as indicators of gaze direction during the trials.

### Procedure

Each participant completed three separate sessions on different days: one baseline and two gazing sessions. Each session began by calibrating the HMD to align the virtual cyclist’s perspective with the participant’s real head position. Next, participants performed a 5-minute self-paced warm-up on the bicycle trainer while immersed in the virtual environment, followed by a 3-minute rest before starting the trial. This procedure remained consistent across all sessions.

#### Baseline session.

The baseline session began with an explanation that the study aimed to investigate the effect of VR on cycling performance and sensations. Participants were informed that they would complete three cycling trials during which we would record their power output. They were told that their goal in this first session was to produce the highest possible average power output for 20 minutes while viewing a VR environment, and that their goal in the subsequent sessions would be similar, with some minor differences.

We then introduced the RPE and PM items. We explained that the RPE item was intended to measure their overall perceived exertion at that moment, providing examples of possible scores from Borg [30]. We clarified that the PM item aimed to assess their perception of PM which we explained beforehand as the feeling that “things are moving in the right direction”, that participants were “on track to succeed”, and that they were “on a roll”. This explanation was based on Vallerand et al.’s [32] conceptualization of PM as the perception of making progress toward a goal, and follows previous instruction protocols of PM research in cycling [21]. Participants were informed that both items would appear every 2 minutes during the trial, at which point they would need to verbally provide a rating from 0 to 10. We emphasized that there were no right or wrong answers, encouraging them to choose a number that best reflected their feelings. Before the trial, participants familiarized themselves with the items. The items first appeared at the 1-minute mark and then at 2-minute intervals thereafter. The order of the items was counterbalanced, with the first item disappearing after the first response, followed immediately by the second item.

After these explanations, participants completed the warm-up, followed by a short break, and then performed the baseline trial. During this trial, they wore the HMD but no gazing frames were present in the virtual environment. Upon completion, they were informed of their average power output and told that the goal for the next two sessions would be to surpass this baseline power level.

#### Proximal and distal gazing sessions.

In the second and third sessions, we introduced the experimental conditions before the trial. We demonstrated both the proximal and distal frames and asked them to position themselves in a way in which they could see both comfortably. Before starting the trial, we reminded participants of their average baseline power output and emphasized the goal of surpassing it. Following these explanations, participants completed the trial in either the proximal or distal condition, in counterbalanced order, with the RPE and PM items appearing every 2 minutes. Participants were instructed to keep their gaze within the frame throughout the trial, and we emphasized that this was crucial for the study. We consistently had two researchers present in the experimental room who could observe participants’ gaze direction on a desktop monitor and if participants appeared to look outside the frame, they were reminded to maintain their gaze within the instructed area. This happened once with a participant who briefly looked away from the frame. In the second gazing trial, participants were not informed of their performance in the first gazing trial. At the end of the third session, they received their results and were debriefed about the experiment.

### Data analysis

Exerted effort, RPE and PM scores, and eye-tracking data were collected in Microsoft Excel where averages per trial were calculated before statistical analysis. All statistical analyses were conducted using SPSS 29.0.1 (IBM Corp, Armonk, NY, USA), except for the repeated measures correlation analysis, which was performed using rmcorrShiny [34].

As a first check, we analyzed the eye-tracking data to determine whether participants directed their gaze higher and farther away in the distal condition than in the proximal condition. Specifically, we assessed the average vertical and longitudinal coordinates of the hitpoints in each condition to evaluate differences in gaze direction. For the vertical coordinate, we used its absolute value as recorded in the scene. For the longitudinal coordinate, we calculated the difference between the hitpoint and the gaze origin coordinate. This adjustment ensured that we measured gaze distance relative to the cyclist’s current location rather than its absolute position within the scene, which would naturally increase as the cyclist moved forward through the virtual environment. We conducted two repeated-measures ANOVAs: one to compare the vertical gaze coordinate and another to examine the longitudinal gaze coordinate.

Next, for each trial, we calculated the average exerted effort based on power output, measured in Watts. Conforming to Otten et al. [7], to investigate the effect of gaze conditions on exerted effort, we first performed a one-way repeated measures ANCOVA, including baseline power output as a covariate to account for differences in cycling levels among the participants.

Lastly, to explore effects on RPE and PM, we conducted one-way repeated measures ANOVAs to examine how gaze conditions influenced participants’ average RPE and PM experience. Additionally, we conducted a repeated measures correlation analysis [34] to explore the relationship between PM and exerted effort.

## Results

### Gaze coordinates

A repeated measures ANOVA with condition as the within-subjects factor and average vertical gaze coordinate as the dependent variable showed a significant difference in vertical gaze coordinate between the proximal and distal conditions with a very large effect size, *F*(1, 27) = 10.00, *p* = .004, *η*_*p*_² = .27. Participants gazed significantly lower in the proximal condition (*M* = 1.29, *SD* = 0.24 meters) than in the distal condition (*M* = 2.43, *SD* = 0.32 meters). Similarly, a repeated measures ANOVA with condition as the predictor and average longitudinal gaze coordinate as the dependent variable revealed a significant difference in longitudinal gaze coordinate between conditions with a very large effect size, *F*(1, 27) = 15.64, *p* < .001, *η*_*p*_² = .37. Participants gazed significantly more nearby in the proximal condition (*M* = 7.49, *SD* = 2.03 meters) than in the distal condition (*M* = 16.28, *SD* = 1.38 meters).

### Exerted effort

**Fig 2:**
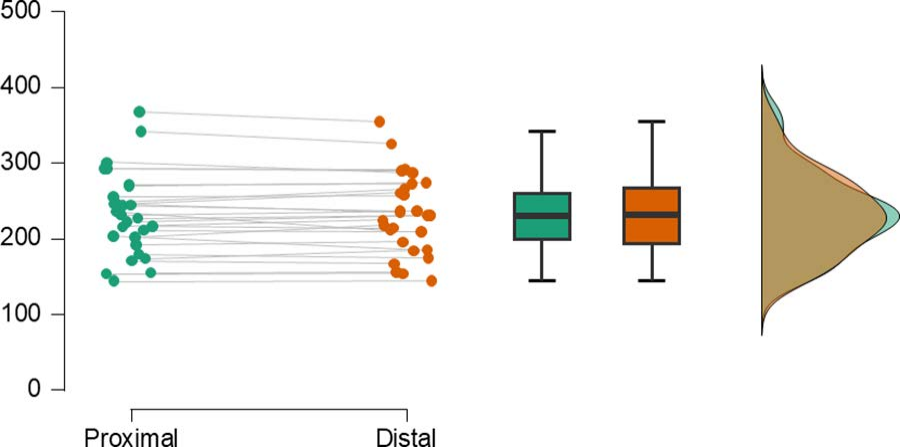
Distribution of exerted effort values in the proximal and distal gaze conditions.

Fig 2 shows the distribution of exerted effort in the proximal and distal gaze conditions. A repeated measures ANCOVA, with condition as the within-subjects factor, baseline power (*M* = 234.21, *SD* = 53.19 W) as a covariate, and exerted effort as the dependent variable, showed no significant main effect of condition, *F*(1, 26) = 0.71, *p* = .407, *η*_p_² = .03, and no significant interaction effect between condition and baseline power, *F*(1, 26) = 0.93, *p* = .343, *η*_p_² = .04. Exerted effort was on average 232.82 W (*SD* = 54.71) in the proximal condition and 231.95 W (*SD* = 52.37) in the distal condition.

### Perceived effort and psychological momentum

A one-way repeated measures ANOVA showed no significant effect of condition on average RPE, *F*(1,27) = 0.467, *p* = .500, *η*_p_² = .02 (proximal condition: *M* = 6.37, *SD* = 1.12; distal condition: *M* = 6.28, *SD* = 1.14). Similarly, a one-way repeated measures ANOVA showed no significant difference between the conditions in PM experience, *F*(1,27) = 0.498, *p* = .486, *η*_p_² = .02, with an average PM score of 5.78 (*SD* = 1.39) in the proximal condition and 5.62 (*SD* = 1.69) in the distal condition. Additionally, a repeated measures correlation analysis showed no significant correlation between PM and exerted effort, *r*_rm_(27) = 0.00, *p* = 0.999.

## Discussion

With this study we aimed to test whether gazing toward proximal or distal areas of the environment affects exerted effort during cycling trials. Building on previous findings that cycling effort was influenced by exposure to proximal and distal areas, and that this relationship depended on cyclists’ baseline power [7], we aimed to test whether similar effects could be found by guiding gaze direction toward these areas. By guiding the gaze through virtual frames instead of occluding areas, we aimed to replicate these findings in a more naturalistic setting, allowing both proximal and distal areas to remain present in cyclists’ peripheral vision. Our hypothesis was that cyclists would exert more effort when gazing at proximal areas than when gazing at distal areas. If this were the case, we aimed to explore whether this effect would depend on baseline power. While average differences were in the expected direction, the results did not provide statistical support for our hypothesis. We observed no significant differences in exerted effort between the proximal and distal conditions, nor a significant interaction effect between gaze condition and baseline power output. Hence, using guided gazing instead of visual occlusion, we did not replicate the effects reported by Otten et al. [7].

A possible explanation for this finding is that exposure to proximal and distal areas through occlusion, as employed by Otten et al., may not be translatable to the effects of proximal and distal gazing. With the proximal and distal areas remaining present in peripheral vision, cyclists might not experience the same change in optic flow velocity as they would with occlusion. As the retinal periphery can effectively process motion [28,29], participants directing their gaze toward proximal areas could still process motion from distal areas and vice versa, which may have reduced the influence of our gaze manipulations. In contrast, visual occlusion removes peripheral motion, which could explain why Otten et al. observed stronger effects on exerted effort. This interpretation aligns with prior work highlighting the role of FoV restrictions in the effects of optic flow manipulations on speed perception, which showed that reducing peripheral vision leads to lower perceived speed [23–27].

That said, Banton et al. [6] found that perceived speed was about 50% slower when gazing straight ahead compared to when looking downward, which initially led us to suggest that gaze direction alone could influence speed perception and, consequently, exerted cycling effort. However, Banton et al.’s findings can also be interpreted in light of FoV restrictions. In VR, altering gaze direction does not equate to altering gaze direction in real life. That is, although immersive, VR presented through HMDs presents a smaller part of the visual environment than natural vision. For instance, Banton et al. used an HMD with a 52° diagonal FoV, which is about one third of the natural FoV. This means that gazing straight ahead still limits the amount of peripheral optic flow available, potentially contributing to the effect of gaze direction on speed perception. In contrast, our study used a 110° diagonal FoV, which, while still more restrictive than natural vision, allowed more peripheral motion to remain visible and may have reduced the impact of gaze direction on exerted effort.

Overall, while previous research has shown that cycling effort is influenced by the optic flow that relates to one’s movement through the environment [4,7], our findings suggest that cyclists may not be able to use this to their advantage by adjusting their gaze. Specifically, we were not able to demonstrate that gazing toward areas with faster flowing elements does offer performance benefits. Although one might speculate that occluding other areas of the visual field could enhance such effects, this may only be possible within controlled experimental environments, not in real-world settings. This further underscores the capacity of VR and its highly controllable nature to impact effort regulation [3,35,36].

## Limitations

While we did not find statistical evidence supporting the hypothesized effects of gaze direction on exerted cycling effort, it is important to interpret these results with caution. With 28 participants, our study had a statistical power of.72, meaning it was moderately powered to detect medium to large effects but may have lacked sensitivity to identify smaller but meaningful effects. Moreover, the differences in average exerted effort, as well as in the exploratory measures of RPE and PM experience, were in the hypothesized direction with cyclists exerting slightly more effort and reporting slightly higher RPE and PM in the proximal condition than in the distal condition. Future studies with larger sample sizes may be better equipped to identify small but meaningful effects of gaze direction on these measures.

Another methodological challenge was accurately tracking gaze direction throughout the cycling trials. The eye-tracking system struggled to maintain accuracy during the 20-minute cycling sessions due to an increasing amount of sweat on the camera lenses. This led to increasingly unreliable gaze data and only rough approximations of where participants were actually looking. Although the results of our analyses indicate that participants gazed significantly higher and farther away in the distal condition compared to the proximal condition, we cannot verify based on the data whether participants consistently gazed within the frames throughout the trials. However, in addition to the rougher estimates based on the gaze data, we had two researchers present in the experimental room who could observe participants’ gaze direction on a desktop monitor, and no consistent deviations from the gaze instructions were observed.

## Conclusions

In conclusion, this study aimed to examine whether gaze direction toward proximal or distal areas affects exerted effort during cycling. However, we did not find statistical evidence that gaze direction influences exerted effort. These findings suggest that the effects of guided gazing through virtual frames, while allowing both proximal and distal areas to remain visible in the peripheral field, may have a less pronounced effect than visual occlusion [7]. From a practical standpoint, this raises questions about whether gaze direction meaningfully influences cycling performance, especially when both proximal and distal areas remain visible.

From a scientific standpoint, our results suggest that the retinal periphery’s ability to process motion may have diminished the influence of gaze direction on exerted effort and that the larger FoV may have reduced the effects of these gaze manipulations. Future research could investigate how varying the FoV influences the relationship between gaze direction and exerted effort. For example, examining a broader range of FoV sizes could clarify whether a more restricted visual field increases the effects of gaze direction, while a more expansive field reduces them. Understanding this relationship could provide valuable insights into how (peripheral) vision affects cycling effort in both virtual and real-world environments.
